# Biobanking in East and Central Africa: A case of the Integrated Biorepository of H3Africa Uganda

**DOI:** 10.12688/openresafrica.13495.1

**Published:** 2022-09-30

**Authors:** Gideon Nsubuga, David Patrick Kateete, Sharley Melissa Aloyo, Lwanga Newton Kigingi, Nasinghe Emmanuel, Kezimbira Dafala, Moses Levi Ntayi, Moses L Joloba, Kamulegeya Rogers

**Affiliations:** 1 Integrated Biorepository of H3Africa Uganda, Makerere University, Kampala, P.O. BOX 7072, Uganda; 2 Department of Immunology and Molecular Biology, School of Biomedical Sciences, College of Health Sciences, Makerere University, Kampala, P.O. BOX 7072, Uganda

**Keywords:** Biobanking, East Africa, Central Africa

## Abstract

Biorepositories are essential because they guarantee the proper storage and distribution of biospecimens and their associated data for current and future research. In Eastern and Central Africa, the Integrated Biorepository of H3Africa Uganda (IBRH3AU) at Makerere University in Uganda was the first of its kind. It is strategically located at Makerere University College of Health Sciences, which is home to some of Uganda's most relevant and impactful infectious and non-infectious disease research.  Since its inception as a pilot project in 2012, the IBRH3AU biorepository has grown into a state-of-the-art facility serving the H3Africa consortium and the rest of the scientific community. IBRH3AU has built a solid infrastructure over the past ten years with cutting-edge methods and technologies for the collection, processing, quality control, handling, management, storage and shipment of biospecimens. H3Africa researchers, local researchers, postgraduate and postdoctoral students, and the greater scientific community in Eastern and Central Africa and beyond have benefited from IBRH3AU's exceptional biobanking services.

## Disclaimer

The views expressed in this article are those of the authors. Publication in Open Research Africa does not imply endorsement by F1000 or our partners.

## Background

Africa is disproportionately affected by communicable and non-communicable diseases ^1– 5^. This disease burden results from complex interactions between genes, the environment, and lifestyle ^6^. Africans have the most genetic diversity ^7, 8^, yet little is known about how genes and the environment affect disease in Africa ^9^. Contextually, 96% of the available genomic data is derived from non-Africans ^10^. As such, our current understanding of the interplay between genetics and disease is biased ^9, 11^. The Human Heredity and Health in Africa (H3Africa) Consortium set out to shift the narrative by increasing genomic research capability and infrastructure ^12^. Biorepositories, among other approaches, have been established to achieve this.

Biorepositories are crucial because they ensure that biospecimens are properly stored and disseminated appropriately for current and future research ^13^. Since the outset, H3Africa has emphasized the importance of biorepositories in developing Africa's indigenous capacity for genomic research ^14^. In this regard, the National Institutes of Health-funded H3Africa Program established biorepositories in Uganda, South Africa, and Nigeria between 2012 and 2013 ^15^. The biorepositories were created so that scientists all around the world could retrieve and access well-kept, well-annotated biospecimens from research projects in the H3Africa Consortium Program.

The Integrated Biorepository of H3Africa Uganda (IBRH3AU) at Makerere University in Uganda is the first of its kind in Eastern Africa ^15, 16^. It is strategically located at Makerere University College of Health Sciences, which is home to some of the country's most relevant and impactful research in infectious and non-infectious diseases. Makerere University College of Health Sciences is affiliated with the Mulago National Teaching and Referral Hospital, which sees over half a million patients annually, and the biorepository, by extrapolation, has access to this niche of biospecimens. Collectively, the biorepository has become a valuable source of high-quality samples for researchers studying infectious and non-communicable diseases in African populations both within and outside the country, with over 500,000 samples in storage ^17^. IBRH3AU is a cutting-edge, world-class biorepository with an expanded capacity for collecting, processing, and storing samples for scientists conducting genomic research in Africa and elsewhere. Notably, because the H3Africa Consortium supports the IBRH3AU, its operations are subject to the Consortium's rules and regulations.

IBRH3AU has evolved over the years from a pilot project to a world-class, state-of-the-art facility that provides biorepository services to the H3Africa consortium and the rest of the scientific community. Over the past ten years, IBRH3AU has built a solid infrastructure with cutting-edge methods and technologies for biospecimen collection, processing, quality control, handling, management, and storage. IBRH3AU's exceptional biobanking services have supported research and training for H3Africa researchers, local researchers, postgraduate and postdoctoral students, and the greater scientific community in Eastern and Central Africa and beyond.

## Organization and structure

The Principal Investigator (PI) received funding from the US National Institutes of Health, grant number UH 5U24HG007051 of the H3Africa Common Fund Initiative to establish the biorepository for Eastern and Central Africa (IBRH3AU). IBRH3AU has an advisory board, a steering committee, and a staff structure. The IBRH3AU's functions are overseen by an advisory board. This multidisciplinary board includes representatives from the Ministry of Health, Makerere University, and the community. The steering committee, which is in charge of the biorepository's day-to-day operations, receives strategic direction from the advisory board. IBRH3AU is led and guided by a Steering Committee that consists of the Principal Investigator, Co-Investigators, Biorepository Coordinator, ELSI Officer, Business Manager, and Project Administrator. The Biorepository is managed by the Biorepository manager, who leads a team of laboratory scientists, a nurse, data managers, and volunteers as they come in.

## Ethical, legal, and social issues

The IBRH3AU project was approved by the Uganda National Council for Science and Technology and the School of Biomedical Sciences Research Ethics Committee (SBS-326) at Makerere University College of Health Sciences. IBRH3AU has been able to maintain annual approvals for the last ten years. All bio-specimens are collected and shared according to ethical and legal requirements. In addition, all research studies that deposit samples in a biorepository are required to have an IRB-approved study protocol. Material transfer agreements (MTAs) are obtained for all biospecimen transfers.

## Establishment of state-of-the-art biobanking facilities

**Figure 1.**
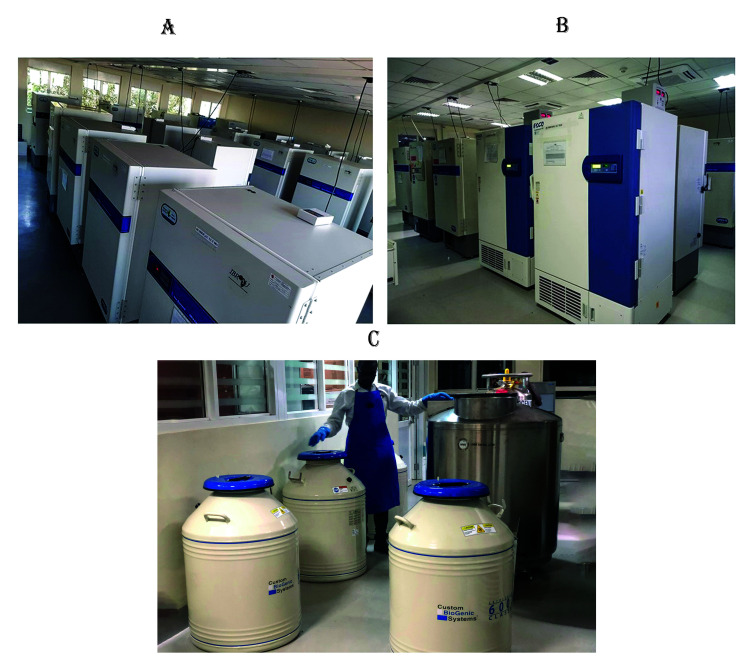
**1A**. and **1B**. show IBRH3AU's Freezer Firm section. **1C**. shows part of the Liquid Nitrogen Room. (Written consent for publication of the image was obtained from the individual included in the image.)

Since its inception as a pilot project in 2012, IBRH3AU has evolved into a local, regional, and global biorepository with a 6,500-square-foot H3Africa facility as shown in Figure 1. IBRH3AU has a liquid nitrogen storage room (LNSR) and a freezer firm section as well as world-class laboratories for immunology, microbiology, molecular biology, and mycobacteriology that serve as supporting laboratories. The LNSR is equipped with ten liquid nitrogen tanks with a capacity of 32,000 2 ml vials as well as the MVE High Efficiency 1500 Series -190°C freezers, which provide cryogenic storage for up to 60,000 2 ml vials. The freezer firm is equipped with 20 ultra-low temperature (ULT) freezers, four refrigerators, and four -20°C freezers. The ULT freezers are capable of storage from -70 to -80°C and have a capacity of 50,400 2 ml vials each. The refrigerators and the -20°C freezers have a capacity of 852 litres each. In addition, IBRH3AU has 300 square feet of room temperature storage. All these storage environments are monitored by a system capable of sending off-site messages to up to four staff members. This monitoring extends to levels of carbon dioxide, oxygen, and humidity within the facility. This equipment runs in an environment with sufficient tasks, and general lighting and temperature are controlled by a heating, ventilation, and air conditioning (HVAC) system and free-opening wide windows as a last resort backup. All electrically powered units are backed up by three robust 200 KVA generators installed in series that are serviced and maintained with enough fuel reservoirs to run for up to 72 hours in case of power failure. All the equipment and the facility are covered by an annual service and maintenance contract. All of the staff are well-trained on how to use the equipment. Standard Operating Procedures (SOPs) are in place, and the staff's skills are constantly being updated. These facilities are monitored with CCTV and have a dedicated power line, and staff perform safety and disaster management drills annually as per our safety manual and disaster management policies.

## Heightened biorepository operations

**Figure 2.**
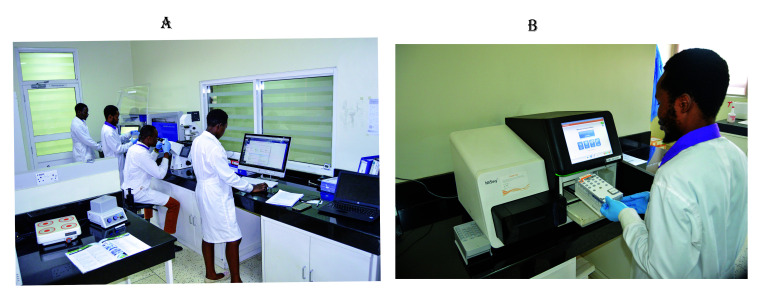
**2A**. shows IBRH3AU staff processing biospecimens in the laboratory. **2B**. shows IBRH3AU staff operating a MiSeq analyser for next generation sequencing. (Written consent for publication of the images was obtained from the individuals included in each image.)

Centralised and standardised biorepository operations have been performed by IBRH3AU for the H3Africa Consortium and other researchers. Biospecimens have been received, processed, quality controlled, stored, and shipped to and from various H3Africa research sites from its area of jurisdiction, i.e., Eastern and Central Africa, as indicated in Table 1, and other researchers as indicated in Table 2. IBRH3AU engages the H3Africa sites under its jurisdiction, namely TrypanoGEN (Uganda, DRC, and Burkina Faso), CAfGEN (Botswana, Swaziland, and Uganda), TB systems biology (Ethiopia), and Rwanda, on a continuous basis to assess the need to transfer biospecimens to the H3Africa biorepository in Kampala. Over the years, IBRH3AU developed a custom programme that has resulted in the biorepository receiving DNA from various research projects in the area. As a result of continuous biobank awareness initiatives for academics and Makerere University faculty members, the IBRH3AU Biobank presently supports over 22 non-H3African researchers, as shown in Table 2. Figure 2 shows the processing of biospecimens at IBRH3AU.

**Table 1.** H3Africa consortium projects and their sample types that are supported by IBRH3AU.

Project name and site location	Sample type
CAfGEN	Plasma
(Uganda & Botswana)	PBMCs
	Nucleic acids - DNA
	Whole Blood (RNA Paxgene tubes)
	DNA (Botswana)
TrypanoGEN	DNA from whole blood
(Uganda, DR Congo & Burkina Faso)	
TBGEN Network Ethiopia	DNA from whole blood
PTSD Rwanda	DNA from whole blood

**Table 2.** Non H3Africa studies and their sample types that are supported by IBRH3AU.

Study CODE	Study title/Name/Location	Status	Sample types
ACCESS	Task Shifting Screening and Measurement of Blood Pressure and Blood Sugars by Community Health Workers for Early Detection of Referral of Hypertension and Diabeted in Rural Uganda	Ongoing	SR, WB
AESA AMR	Understanding Transmission dynamics and acquisition of antimicrobial resistance at Referral Hospitals and community settings in East Africa	Target Reached	TBI, DNA
AFRICA SUITCASE	Validation of Novel diagnostic technologies for the confirmatory diagnosis of emerging infections, including SARS-COV-2	Ongoing	UAS, RNA
ASAP	African Severe Asthma Program	Target Reached	PXD, PXR, SR, WB
ATOB	Advancing Tissue and Organ Tissue in Uganda	Ongoing	CB, SC, CPL, SP
CHAIN	The Childhood Acute Illness & Nutrition Network	Ongoing	SR, PL, ST, WB, RS
COPD	Common Obstructive Pulmonary Disease in Uganda	Ongoing	SP, WB, PL, SR, BAL
COVBANK	Establishment of quality assured COVID-19 specimen repository to support research in diagnosis, prevention and management of SARS-CoV-2 in Uganda	Ongoing	SR, PL, WB, ST, RNA, SL, UR, TS
COVID Sero Survey	A population-based, age- and gender- stratified sero-survey study for SARS-CoV-2	Target Reached	SL, PL, WB, UAS
Curie	Cerebral Palsy in Uganda: Multi-Component Community Based Intervention Programme	Target Reached	SL, DNA
DRUM	Drivers of antimicrobial Resistance in Uganda and Malawi	Ongoing	Pax, SL, ST, DNA Extraction
ELITE	Host genes responsible for T Cell resistance to HIV in Ugandan elite and viremic controllers	Ongoing	WB, PL, SR
FEND-TB	Feasibility of Novel New Diagnostics for TB in Endemic countries	Ongoing	PL, SL, SP, SW, UR, WB, MW, TBI, TBP, UAS, MS
GHU	Golobal Health Uganda	Ongoing	PL, SR, WB
GOAL-Post	GOAL Post (GwokO Adunu pa Lutino, “protect the heart of a child”)	Ongoing	WB, SL, PL
HAP	Household Air Pollution	Ongoing	FL
HPV DNA	Feasibility and acceptability of HPV DNA self-sampling among women attending a rural HIV Clinic	Target Reached	UR, PL, SR
ITL-LAB	Immunology Teaching Laboratory, Makerere University	Ongoing	PBMC, PL, SR, WB, UR
MAGNUS	Milk affecting growth, cognition and the gut in child stunting	Target Reached	SR, PL, ST, WB
MBC	Evaluation of Malaria Infection among school going children in Uganda	Target Reached	DBS, PL, SR
MBL-LAB	Makerere University Molecular Biology Laboratory	Ongoing	DNA, RNA, PR, PL, WBT, UR
MLMU	Microbiology Laboaratory Makerere University	Ongoing	BCT, DNA
MYB	Mycobacteriology Laboratory Makerere University	Ongoing	TBI, TBP, WB, SP, SR, PL, DNA
NCD	MUCHAP/ Iganga Mayuge HDSS - NCD Cohort Feasibility Study	Target Reached	PL, SR, WB.
NEUROGAP	Neuropsychiatric Genetics in African Populations	Ongoing	SL, DNA
NOD-ped TB	Novel and Optimized Diagnostics in Pediatric Tuberculosis in Kampala Uganda	Ongoing	ISL, PL, SL, SP, UAS, UR, WB, ST
PB SAM	Pancreatic Enzymes and Bile Acids: A Non-Antibiotic approach to Treat Intestinal Dysbiosis in Acutely IllSeverely Malnourished Children	Ongoing	RS, ST, PL, WB, ST
PEXADU	Pesticide Exposure, Asthma and Diabetes in Uganda	Target Reached	SB, PL, WB
PHS	Uro Care - Africa Medical and Behavioral Sciences Organization	Ongoing	PL, SR
Predic TB	Validating a clinical risk score for early management of tuberculosis in Ugandan primary health clinics- PredicTB study	Ongoing	SP, TBI, TBP
Predontal	Exploring the Association between periodontal disease and the risk of Alzheimer's disease and related dementias: A comparative study	Ongoing	WB
Pre-FIT	Predicting Future Incipient Tuberculosis	Ongoing	PL, WBT, RNA, UR, TBI
Pulmonomics	Impact of TB and HIV co-infection on host and microbial gene expression in the upper airway	Ongoing	TBI, WB
RAS Survey 1	First COVID-19 Rapid Assessment Survey in Uganda	Target Reached	SL, PL, WB, UAS
RAS Survey 2	Second COVID-19 Rapid Assessment Survey in Uganda	Target Reached	SL, PL, WB, UAS
Stool4TB	Stool Based qPCR for the diagnosis of TB in children and people living with HIV	Ongoing	ST, UR, WB, PXR, PL
SURE	A randomised Trial of 6 Months Intensified Ant-Tuberculosis and 2 Months Anti-inflamatory Treatment for HIV-Infected and HIV-Uninfected African and Asian Children with Tuberculous Meningitis	Ongoing	TBI, TBP, SP
TB-SPEED	TB Speed Pneumonia	Ongoing	NPA, SR, PL, ST, WB, RS
TB-SPEED SAM	TB Speed Severe Acute Malnutrition	Ongoing	SR, PL, ST, WB, RS
TURN-TB	Trace Ultra Results insight in Tuberculosis Screening	Ongoing	WB, PXR, RNA
UBV-01N	A Phase IIa randomized double-blind, placebo-controlled clinical trial to determine the preliminary safety and efficacy of UBV-01N in adult patients infected with SARS-CoV-2 (COVID-19) in Mulago National Referral Hospital	Target Reached	PL, SR, WB.
UCU SASA	Uganda Christian University, Department of Agriculture and Biological Sciences	Ongoing	DNA, PLV, PRT
Urine LAM	Development of Large-Scale Panel of TB Patient Urine & Serum Sample to serve as standards for detection of TB-LAM in HIV-Positive and Negative presumptive TB patients	Target Reached	UR

**KEY:** BAL, Bronchoalveolar Lavage; BCT, Bacteria Culture Isolates; CB, Cord Blood; CPL, Cord Blood Plasma; DBS, Dry Blood Spot; DNA, Deoxyribonucleic acid; FL, Air Pollution Filters; MS, Mouth Swabs; MW, Mouth Wash; ON, Still Ongoing; PL, Plasma; PLV, Plant Leaves; PR, Proteins; PRT, Plant Roots; PXD, PaxGene DNA; PXR, PaxGene; RNA, Ribonucleic acid; RS, Rectal Swabs; SC, Stem Cells; SL, Saliva; SP, Sputum; SP, Sperm; SR, Serum; ST, Stool; TBI, Mycobacterium Tuberculosis Isolates; TBP, Mycobacterium Tuberculosis Pellets; TR, Target Reached; TS, Tissue; UAS, Upper Airway Swab; Urine, Urine; WB, Whole Blood

The collection and processing of biospecimens has been standardised by IBRH3AU, resulting in a streamlined workflow. These efforts have ensured that only high-quality specimens are sent to IBRH3AU, as all submission sites must follow the same protocols and guidelines. IBRH3AU has also created and implemented a strict quality control and quality assurance (QC/QA) programme. It puts its processes through QC/QA from ISBER-endorsed and ISO-certified external quality assurance programmes and internal QC/QA measures, such as internal audits, and uses audit reports to make improvements all the time. External quality assurance (EQA) panels include, but are not limited to, DNA and RNA extraction, quantification and purity of DNA and RNA, and peripheral blood mononuclear cells (PBMC) viability, and IBRH3AU has always obtained good results.

To date, IBRH3AU has successfully prepared and received biospecimen shipments, which can be tracked online at any time during transit using the airway bill numbers provided.

## Strengthening biorepository management and scientific expertise

**Figure 3.**
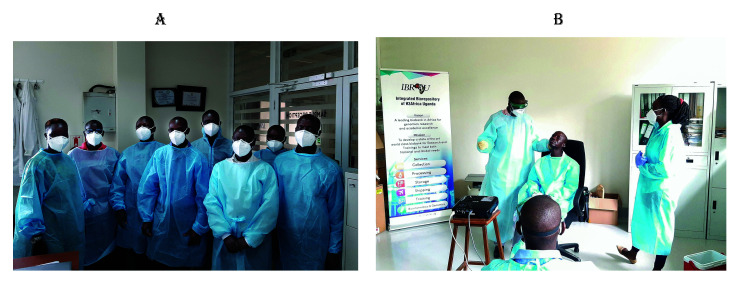
**3A**. shows some of the trainees during the training sessions. **3B**. shows IBRH3AU staff training health personnel in COVID-19 sample collection. (Written consent for publication of the images was obtained from the individuals included in each image.)

IBRH3AU's strategic location within Makerere University's Department of Immunology and Molecular Biology ensures a steady stream of students and visiting researchers drawn to the need for biobanking knowledge. As a result of its years of experience and extensive training in biorepository science and management from the University of Luxembourg, the University of Washington ICRC repository, and George Washington University, IBRH3AU provides training for a variety of biobanking activities, including biospecimen collection, processing, storage, and management. One of IBRH3AU's courses, “MSB 7212 Fundamentals of Biobanking", has been approved as an elective for graduate students studying for a Master of Science in Bioinformatics at the College of Health Sciences, Makerere University. During the COVID-19 pandemic, IBRH3AU trained more than 300 healthcare professionals from more than 20 healthcare centres, laboratories, and hospitals on how to collect, process, and transport COVID-19 biospecimens as shown in Figure 3. IBRH3AU scientists have also contributed to the course "From Swab to Server: Testing, Sequencing, and Sharing During a Pandemic" on the popular online learning platform Future Learn. IBRH3AU has facilitated the training of stem cell researchers, master’s students, and PhD students in cord blood collection and stem cell processing and storage principles as part of an effort to advance Uganda's tissue and organ biobanking. Fifteen Makerere University staff members have been trained in the principles of human sperm collection, processing, and storage as part of the same effort.

## Contributions to shared resources

IBRH3AU made a big contribution to the first edition of Uganda's National Research Biobanking Guidelines, which came out in 2021. Before that, Uganda didn't have any biobanking guidelines. IBRH3AU developed the minimum data fields, forms, and SOPs required for biospecimen shipping and barcoding through collaborations with the various H3Africa biorepositories. The H3Africa catalogue, which is housed at the H3AfricaBionet, receives the developed data fields on a regular basis from IBRH3AU. The IBRH3AU was the driving force behind the creation of the H3Africa Biorepository programme website, which, in collaboration with other biorepositories, H3Africa research sites, and H3Africa Bionet, created a biorepository catalogue that allows online access to well-catalogued biospecimens. IBRH3AU has been a member of the Data and Biospecimen Access Committee (DBAC) working group for many years, helping to develop data and biospecimen access guidelines, forms, policies, and agreements.

## Tissue and organ biobanking advancement in Uganda

Uganda is currently developing its transplant medicine capacity by training healthcare workers and establishing specialised hospitals in both the public and private sectors. But one thing that is still missing in this field is the ability to have quality-assured biobanks that collect, store, retrieve, and distribute viable tissues and organs in a well-regulated, standardised way. Having pioneered biobanking in Uganda, the IBRH3AU’s multidisciplinary team set out to expand its scope into tissue and organ biobanking, starting with sperm, ovum, and umbilical cord blood. IBRH3AU has (a) established the first organ and tissue biobank in Uganda; (b) established a web-based catalogue of donors and recipients; and (c) developed standard operating procedures for the collection, processing, and storage of human organs.

## Uganda's enhanced response to COVID-19

**Figure 4.**
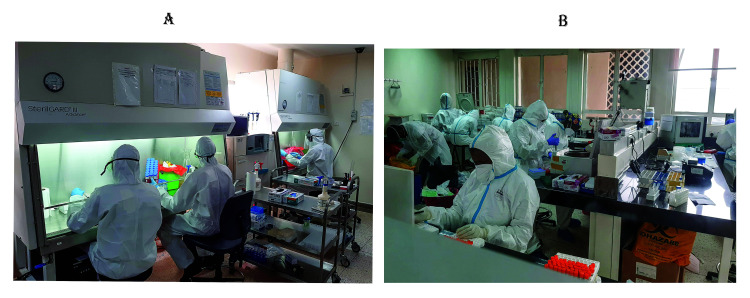
IBRH3AU staff performing COVID-19 sample processing. (Written consent for publication of the images was obtained from the individuals included in each image.)

During the COVID-19 pandemic, IBRH3AU provided significant resources to the Makerere University Rapid Assessment team in terms of consumables, personnel, and laboratory testing. IBRH3AU also established a biobank of well-annotated and well-characterized samples collected from COVID-19 positive patients and negative controls, which has proven to be a valuable resource in Uganda's rapid response to COVID-19 ^16^. With over 100,000 biospecimens in storage, the COVBANK provides researchers and students with high-quality biospecimens and has facilitated the development of population-appropriate diagnostic tools, therapeutics, vaccines, and control measures. Figure 4 shows IBRH3AU staff working on COVID-19 sample processing.

## Conclusions

As contributors to research publications, biorepositories that coordinate biobanking activity rank as one of the most essential medical research infrastructures. H3Africa researchers, local researchers, postgraduate and postdoctoral students, and the greater scientific community in Eastern and Central Africa and beyond have benefited from IBRH3AU's exceptional biobanking services.

## Consent

Written consent for publication of the images was obtained from the individuals included in each image.

## Data Availability

No data are associated with this article.
