# Supplementary material for: Biobanking in East and Central Africa: A case of the Integrated Biorepository of H3Africa Uganda
Source: Open Res Afr. 2022 Sep 30;5:33. [Version 1] doi: 10.12688/openresafrica.13495.1 (PMC10175960; doi:10.12688/openresafrica.13495.1)
Supplement: Peer review history

# Peer review: Reviewer response for version 1

**DOI:** 10.21956/openresafrica.14646.r29634

**Published:** 2023-05-18

**Authors:** Monika Schmelz

**Affiliations:** 1 The University of Arizona, Tucson, Arizona, USA

This is an important paper describing major impressive accomplishments to advance genomic research and improve health in African populations. Some edits will improve the paper:

 1. Section Background: A short explanation on what the H3Africa (Human Heredity and Health in Africa Consortium) is would help the reader. For example: is it a program of the Ugandan government? What is its overall goal? Who are the partners? Is IBRH3AU a project by H3Africa? This is not clearly stated in the background.

 2. The paper would benefit from some re-organization, for example: in the Background section, third paragraph, last sentence: “Notably, because the H3 Africa

 supports the IBRH3AU…”, this belongs to “Governance and Organization”.

- An organizational chart would be of benefit.

3. Abbreviations: what is an ELSI Officer?

 4. Is the IBRH3AU receiving specimens only from Ugandan sites, or also from other African countries and/or other countries? If so, how are “international“ specimens handled in regard to Ethic Committees Reviews and assurance of Human Subject Protection?

 5. “freezer firm” should be corrected to “freezer farm”.

 6. Section “Establishment of state-of-the-art biobanking facilities”:       

- “…world-class laboratories for immunology…” Are these core laboratories? What is their function? Are they supporting clinical trials? Project based? Quality management - proficiency testing and assurance of assays?
- “All these storage environments are monitored…”. What exactly is monitored? Humidity? Temperature anges? Is there an emergency response plan? Is there an “out of work hour” emergency team on call to respond to alarms? This needs better explaining.
- Sentence on Page 4: “This equipment runs in an environment with sufficient tasks…”:  this sentence is not clear. Capacity? What kind of capacity?

7. There should be an upfront explanation about the scope of the IBRH3AU: For example: what are the types of specimen collections such as:

- Project driven specimen collections (PI initiated, clinical trials with specimen research plans), these specimens are reserved for the primary purpose.
- Random specimen collecting, open to all requests?
- Archival pathology tissue (FFPE)?

8. The section “Establishment of state-of the art biobanking facilities” should be divided into 2 sections:

- Establishment of state-of the art biobanking facilities.
- Quality Management Plan/Program: (has the biobank been accredited, or is there a plan towards that, how often are audits performed and who performs them? There should be a short explanation of the types of datafield recorded in association with specimen history). Are there MOPs (Manual of Procedures) in place for clinical trials or PI initiated specimen collections? MOPs determine timelines, types, frequency etc. of specimen collection. MOP drive biobanking SOPs. How does the biorepository ensure that participating sites adhere to MOP. Biorepositories should function as a control checkpoint for the sites.
- Page 6: "…purity of DNA and RNA, and peripheral blood mononuclear cells (PBMC) viability, and IBRH3AU has always obtained good results”:  More details on percentage rates of passing tests would be more informative.

 9. Section “Contributions to shared resources”:

- “Developing of Ugandan guidelines” may belong to Quality Management.

10. There should be a short explanation about who can request specimens, whether there are costs, or for free, what the request process is and a link to the IBRH3AU website.

 11. Abbreviations in Table 2: check urine.

 12.  It would be really interesting to the reader what types of research the IBRH3AU has supported until now, number of specimens dispersed? Number of publications? The success and justification of any biorepository is measured by the scientific output.

Does the article adequately reference differing views and opinions?

Yes

Are all factual statements correct, and are statements and arguments made adequately supported by citations?

Yes

Is the Open Letter written in accessible language?

Yes

Where applicable, are recommendations and next steps explained clearly for others to follow?

Partly

Is the rationale for the Open Letter provided in sufficient detail?

Partly

Reviewer Expertise:

Biorepository science, cell and molecular biology, immunohistochemistry, image analysis, membrane trafficking  of MHCII in lymphoma

I confirm that I have read this submission and believe that I have an appropriate level of expertise to confirm that it is of an acceptable scientific standard, however I have significant reservations, as outlined above.

# Peer review: Reviewer response for version 1

**DOI:** 10.21956/openresafrica.14646.r29633

**Published:** 2023-05-11

**Authors:** Zisis Kozlakidis

**Affiliations:** 1 World Health Organization, International Agency for Research on Cancer, Lyon, France

- The background section mentions how many samples are in storage. However, there is no indication as to how many samples have been used in the decade since the creation of this biobank. There is information on the studies supported in the tables but no indication as to the relative quantity of samples utilized.
- In the organization structure section, it is unclear what the exact relation of the IBRH3AU is with the other healthcare and educational institutions. The strategic physical location is clear, but does it form part of the local university/hospital or is it a completely separate organization?
- In the ELSI section, could you please specify if the collection according to ethical and legal requirements means 'broad consent' or 'specific consent'?
- In the section 'contributions to shared resources', this is very well described, but is lacking specificity - could you please provide if the contribution to the catalogue includes tens, hundreds or thousands of samples' information? An indicative amount is sufficient to demonstrate the level of contribution.
- In the section 'Tissue and organ biobanking' could you please state the horizon for this activity? Within the next year/5 years/decade?
- There is no mention on the numbers of publications that have resulted from this infrastructure. There is a brief mention that publications have resulted - no references provided. This should be part of the scientific expertise section.
- The conclusion is short and does not include any of the next steps that are planned.
- The interaction with the other H3Africa biobanks is unclear. If there is no interaction that is sometimes possible, and has to be stated. 
- The overall development of biobanking in Uganda can be better presented. This is a major biobank, but not unique and details for some of the other facilities can be provided at a minimum as references.
- Please provide a reference for the first edition of Uganda's National Research Biobanking Guidelines.

Does the article adequately reference differing views and opinions?

Partly

Are all factual statements correct, and are statements and arguments made adequately supported by citations?

Yes

Is the Open Letter written in accessible language?

Yes

Where applicable, are recommendations and next steps explained clearly for others to follow?

Yes

Is the rationale for the Open Letter provided in sufficient detail?

Partly

Reviewer Expertise:

Biobanking and microbiology

I confirm that I have read this submission and believe that I have an appropriate level of expertise to confirm that it is of an acceptable scientific standard, however I have significant reservations, as outlined above.

# Peer review: Reviewer response for version 1

**DOI:** 10.21956/openresafrica.14646.r29631

**Published:** 2023-05-11

**Authors:** Dominique Anderson, Carmen Swanepoel

**Affiliations:** 1 University of the Western Cape, Cape Town, South Africa; 2 Division of Haematology, Department Pathology, National Health Laboratory Services and faculty of medicine and Health Sciences (FMHS), Stellenbosch University, Stellenbosch, South Africa

Biobanking in East and Central Africa: A case of the Integrated Biorepository of H3Africa Uganda was easy to read, logical and highlighted several critical aspects of biorepository science. The letter was not solely focused on the infrastructure, but also the governance, ethics, standardization efforts and education. It is well noted that the biorepository has expanded its capacity to facilitate collection and storage of a multitude of samples and additionally be responsive to emerging needs. The paper was a joy to review and very appropriate for Open Research Africa. The authors give an excellent overview on their biorepository setup and the importance of it in research development within Uganda and beyond. The review was concise, to the point and the paper itself was well written and logical.

 Some suggestions:

Consistency of nomenclature:

 The words 'biospecimen' and 'sample' are used interchangeably. Be consistent and either use one of the other.

Confirm word usage:

 Is the word ‘firm’ correct when referring to the infrastructure, or is the correct term ‘farm’? If the latter, this would need to be changed in text and in the legend for figure 1.

 Pg 3: Background section, line 9: "The Human Heredity and Health in Africa (H3Africa) Consortium set out to shift the narrative by increasing genomic research capability and infrastructure" (add within Africa)

 Pg 3, Background section, 3 ^rd^ paragraph, last sentence: "Notably, because the H3Africa Consortium supports the IBRH3AU, its operations are subject to the Consortium’s rules and regulations" (add as well as local and national regulations).

 Pg 3, Organization and structure section, line 5: "IBRH3AU has an advisory board, a steering committee, and a staff structure" – Comment:  This refers to the local governance but what about the external governance? Does H3Africa have their separate steering committee and biospecimen access committee that advise on biospecimen access to H3Africa researchers and beyond?

 Pg 3, ELSI section:  This focuses on the regulatory compliance related to ethics and sample access and transport. No mention regarding Uganda Privacy protection law and how that will have an impact on current and future biorepository and clinical research functions. I think it would be essential to at least bring it up especially since there is mention of export or rather cross border flow of these valuable data. In addition, in this section, material transfer agreements are mentioned, and I am curious as to whether data transfer agreements are also included.

 Pg 4, Facility section: What about liquid CO2 backup as an additional backup system in case of power and generator failure?

 Pg 4, Heightened biorepository operations section, line 7. Replace the word ‘researchers’ with ‘research initiatives'. Similarly, for the word ‘researcher’ in line 18 on the same page, replace with ‘research initiatives’.

 Pg 6 - ISO-certified external quality assurance is mentioned. Which type?

 Pg 7. In the section Contributions to shared resources, change ‘IBRH3AU made a big contribution to the first edition of Uganda’s National Research Biobanking Guidelines, which came out in 2021. Before that, Uganda didn’t have any biobanking guidelines.’ To ‘IBRH3AU made a big contribution to the first edition of Uganda’s National Research Biobanking Guidelines, which was first published in 2021. Prior to this, Uganda didn’t have any biobanking guidelines.’

- What type of biorepository does it identify with? Population based? Disease specific?
- What data management operations/infrastructure are in place?
- Aside from these comments/suggestions to take into consideration for future use to improve the paper but also to help expand the bioresource, no other major issues were observed with the paper.
- We recommend this paper be accepted for indexing.

Does the article adequately reference differing views and opinions?

Yes

Are all factual statements correct, and are statements and arguments made adequately supported by citations?

Yes

Is the Open Letter written in accessible language?

Yes

Where applicable, are recommendations and next steps explained clearly for others to follow?

Yes

Is the rationale for the Open Letter provided in sufficient detail?

Yes

Reviewer Expertise:

NA

We confirm that we have read this submission and believe that we have an appropriate level of expertise to confirm that it is of an acceptable scientific standard.

# Peer review: Reviewer response for version 1

**DOI:** 10.21956/openresafrica.14646.r29420

**Published:** 2022-11-22

**Authors:** Divyesh Kukadiya

**Affiliations:** 1 Children's National Hospital, Washington, DC, USA

To showcase a global value of the repository, can the authors provide data (in the form of a table) to show samples and data sharing with number of countries and type of samples and data?

 On Page 7, the authors explain the repository’s help with resources in response to COVID pandemic. If the repository has also helped with other major events (e.g, EBOLA or other outbreaks), it would be a good idea to briefly mention it. If the repository has received any endorsement, any past/current accomplishments/success, it would be worth to mention such things as well.

 Would it be worth to mention where is the next comparable repository? That way, the audience would realize the significant value of IBRH3AU.

 Should the authors provide a graph to show the trend of amount and type of samples processed by the repository over the years? That might give a good impression and visibility to the audience.

 Since the repository is large and handles so many samples (types of samples), would it be a good idea to showcase a tree chart to list the number of team members (including leadership and volunteers)? 

 Supply: Should the authors mention from where the supplies (equipment, sample processing machines, tubes, etc.) come from?

 Under the background section (1 ^st^ paragraph), should the following sentences be reworded?

 “The Human Heredity and Health in Africa (H3Africa) Consortium set out to shift the ( *‘this’* instead of *‘the’*?) narrative by increasing genomic research capability and infrastructure. Biorepositories, among other approaches, have been established to achieve this.”

 Under Ethical, legal and social issues:

- IBRH3AU has been able to maintain annual approvals for the last ten years. Should this sentence end as “since the inception of the repository” instead of "for the last ten years"?
- All bio-specimens are collected and shared according to ethical and legal requirements. Should the authors add ‘data’ (in addition to bio-specimens)?

Under Establishment of state-of-the-art biobanking facilities section:

- Please clarify what type of equipment is referred to for “This equipment runs in an environment with sufficient tasks, and,,,”.

Table 1: would it be a good idea to mention month and/or year (since when) the H3Africa consortium projects initiated?

 Under the Heightened biorepository operations section:

- Please confirm if the sentence is referring to DNA or DNA sample for “Over the years, IBRH3AU developed a custom programme that has resulted in the biorepository receiving DNA from various research projects in the area.”

Table 2:

 Please confirm spelling in the title of:

- ACCESS (Diabeted OR Diabetes)
- GHU (Golobal OR Global)?
- Sample type Keys need to be updated. I was not able to find full form for SW, NPA.

Under the conclusion section:

-  Should the authors provide any future plans (improvements, expansion, adding more locations, staff) of the repository?

Does the article adequately reference differing views and opinions?

Yes

Are all factual statements correct, and are statements and arguments made adequately supported by citations?

Yes

Is the Open Letter written in accessible language?

Yes

Where applicable, are recommendations and next steps explained clearly for others to follow?

Not applicable

Is the rationale for the Open Letter provided in sufficient detail?

Yes

Reviewer Expertise:

Over 13 years of experience in Clinical research with research samples processing, storing, shipping/handling experience.

I confirm that I have read this submission and believe that I have an appropriate level of expertise to confirm that it is of an acceptable scientific standard.
